# Toceranib phosphate (Palladia) for the treatment of canine exocrine pancreatic adenocarcinoma

**DOI:** 10.1186/s12917-021-02978-8

**Published:** 2021-08-11

**Authors:** Margaret L. Musser, Chad M. Johannes

**Affiliations:** College of Veterinary Medicine, Iowa State University, 1809 S. Riverside Dr, Ames, Ames, IA 50011 USA

## Abstract

**Background:**

Canine pancreatic carcinoma is a rare, aggressive tumour that is often diagnosed late in the course of disease. Effective treatment strategies have been elusive, and overall survival time is short. In humans, treatment with tyrosine kinase inhibitors alone, or in combination with IV gemcitabine, have been moderately effective. As canine and human pancreatic carcinomas share many clinical aspects, strategies that mimic human treatment regimens may confer a better outcome in canine patients. The aim of this study was to assess the role of the veterinary tyrosine kinase inhibitor, toceranib phosphate, in the treatment of cytologically or histologically confirmed canine pancreatic carcinomas.

**Results:**

Retrospectively, medical records of dogs with confirmed pancreatic carcinoma treated with toceranib were reviewed. Eight dogs were identified that fit the inclusion criteria. Toceranib was well-tolerated by all patients. Six were treated in the gross disease setting. Four had image-based evaluation of clinical benefit (complete response, partial response, or stable disease of > 10 weeks). Of those patients, 1 achieved a partial response, 2 stable disease, and 1 had progressive disease, for an overall clinical benefit rate of 75 %. An additional dog had clinically stable disease that was not confirmed via imaging. The toceranib-specific median overall survival time was 89.5 days (range: 14–506 days).

**Conclusions:**

Although limited in patient number, this small study suggests that toceranib may have biologic activity in dogs with pancreatic carcinoma. Larger, prospective studies are needed to confirm these preliminary results and define the use of toceranib in the microscopic disease setting.

## Background

Canine pancreatic carcinomas are rare, accounting for 0.01–0.07 % of all neoplasia diagnosed in dogs [1, 2]. They typically occur in older dogs of either sex, with Airedales appearing to be at increased risk [1, 2]. Clinical signs associated with pancreatic carcinomas are nonspecific and varied, most commonly including anorexia, vomiting, and abdominal pain. Frequent bloodwork abnormalities include elevations in alkaline phosphatase, alanine aminotransferase, amylase and lipase, and an inflammatory leukogram [3]. Histologically, most canine pancreatic carcinomas are of acinar origin [2]. A recent retrospective study in dogs indicated that pancreatic carcinomas are aggressive, with up to 78 % of patients presenting with overt metastatic disease at diagnosis, most commonly to the liver or regional lymph nodes. Dogs also appear to have a short survival time after diagnosis (mean overall survival time reported: 8 days) [3]. Effective treatment options are limited for dogs diagnosed with pancreatic carcinoma. Options include surgical removal and chemotherapy, although efficacy of systemic chemotherapy has not been definitively proven [3].

Human pancreatic carcinoma is usually ductal in origin [4]. However, it shares clinical features with canine pancreatic carcinoma: detection is usually late in the course of disease, when metastasis is already present, and carries a poor clinical prognosis [5]. Treatment options for human pancreatic carcinoma typically include surgery, chemotherapy, radiation therapy, and targeted therapy, depending on the stage of disease and resectability of the primary mass [6]. Radiation therapy has been used conditionally in the adjuvant and neoadjuvant settings, and for definitive treatment when surgery is not possible, but is complicated by significant adverse events including nausea, vomiting, diarrhoea, and fatigue [7]. Chemotherapy options are extremely limited, with single-agent gemcitabine the only first-line drug approved for advanced pancreatic cancer, and combination chemotherapy regimens have not resulted in significantly improved survival [8]. Thus, alternative treatment options that are more targeted and may be more effective are needed.

Elevated expression of several tyrosine kinase receptors and/or their activating ligands have been found in human pancreatic carcinoma and are associated with a worse clinical outcome. These include epidermal growth factor receptor (EGFR/HER-1/ErbB-1) [9], vascular endothelial growth factor (VEGF) and its receptor (VEGFR) [10], and platelet derived growth factor (PDGF) and its receptor (PDGFR) [11]. Tyrosine kinase inhibitors (TKIs) directed against EGFR, including erlotinib and lapatinib, have shown modest activity against human pancreatic carcinoma. Clinical trials are on-going for TKIs targeting VEGFR (vatalanib, vandetanib, and axitinib) and PDGFR (imatinib). And in 2011, sunitinib (VEGFR and PDGFR TKI) received approval for the treatment of pancreatic neuroendocrine tumours [12].

Toceranib phosphate is a veterinary TKI that is similar to sunitinib, blocking VEGFR and PDGFR, among other tyrosine kinase receptors [13]. Given the observed response to sunitinib in humans with pancreatic cancer, which appears to be biologically similar to canine pancreatic cancer [3], it seems reasonable to expect a biologic response to toceranib in our canine patients. This retrospective study aimed to determine if a biologic response to toceranib is present in dogs with pancreatic carcinoma. Based on the available human literature, and the accepted mechanism of action of toceranib, we hypothesized that toceranib would result in a biologic response when used for inoperative or metastatic pancreatic carcinoma.

## Results

Nine (9) cases were collected via the American College of Veterinary Internal Medicine (ACVIM) listservs spanning 5 years (2015–2020) from 6 institutions. One was excluded due to lack of toceranib use, leaving 8 for data analysis. This population had a mean weight of 19.3 kg (range: 5.3 to 26.4 kg) and a mean age of 9.75 years (range: 5.5 to 12 years). Additional population characteristics are outlined in Table 1. Four patients had concurrent diseases including arthritis, degenerative joint disease, cystoliths, a previous cranial cruciate ligament repair, allergic skin disease, hypothyroidism, hepatic cholangiocarcinoma, degenerative valve disease, idiopathic head tremor, food allergies, and blindness. Medications for these conditions included carprofen, pimobendan, oclacitinib, benazepril, denamarin, methocarbamol, levothyroxine, and gabapentin.

**Table 1:** Patient Characteristics of Dogs Diagnosed with Pancreatic Carcinoma Treated with Toceranib Phosphate

Case Number	Breed	Gender	Diagnosis Via	Toceranib Dose (mg)	Best Response	Imaging used to assess response	Toceranib-specific Survival Time (days)	Cause of Death
1	Golden Retriever	MC	Cytology	2.2	PD	Clinical response	52	Pancreatic Carcinoma
2	Golden Retriever	FS	Cytology/Histopathology	2.8	SD	AUS and CXR every 8–12 weeks	302	Pancreatic Carcinoma
3	Beagle	FS	Histopathology	2.7	*	N/A	LTF: 82	Lost to follow-up
4	Beagle	FS	Histopathology	2.4	*	N/A	14	Pancreatic Carcinoma
5	Shih Tzu	FS	Cytology	2.8	PR	AUS	97	Lost to follow-up
6	Pitbull	MC	Histopathology	2.3	SD	AUS	Alive at Data Capture (05/12/2021): 506	N/A
7	Old English Sheepdog	FS	Cytology	2.2	PD	AUS	17	Unknown
8	Irish Terrier	FS	Histopathology	2.5	SD	Clinical response	301	Pancreatic Carcinoma

*AUS* Abdominal Ultrasound, *CXR* Chest radiographs, *FS* Female spayed, *LTF* Lost to follow-up, *MC* male castrated, *PD* progressive disease, *PR* partial remission, *SD* stable disease

*Toceranib used in the microscopic disease setting

Clinical signs at diagnosis were non-specific and most commonly included vomiting or regurgitation (50 %), anorexia and weight loss (25 %), and increased liver enzymes (25 %). One patient had no clinical signs, the pancreatic carcinoma was an incidental finding during a routine annual abdominal ultrasound for undisclosed reasons.

The mass was noted to be in the right lobe (*n* = 3), left lobe (*n* = 1), body (*n* = 2), both the left lobe and body (*n* = 1), or a diffuse thickening (*n* = 1) of the pancreas. In 6 dogs where the pancreatic mass could be adequately measured (patients with non-surgical gross disease), the mean longest diameter was 3 cm (range: 1–7 cm). Measurements of the mass were most commonly completed via external imaging (abdominal ultrasound or CT scan). Staging tests included chest radiographs (*n* = 5), abdominal ultrasound (*n* = 5), and CT scan (*n* = 1). All but one dog was evaluated for metastatic disease at presentation. In the remaining 7 dogs, 5 had evidence of metastatic disease (71 %), most commonly to the liver and lymph nodes.

Two patients were treated with definitive treatment prior to toceranib use. These patients both received surgery. No patients received IV chemotherapy, metronomic chemotherapy, immunotherapy, or radiation therapy prior to toceranib. Toceranib was used for non-surgical gross disease (*n* = 6) or microscopic disease after surgery (*n* = 2). Toceranib was administered at a mean of 2.5 mg/kg (range: 2.2 to 2.8 mg/kg) 3 days per week (*n* = 7) or every-other-day (*n* = 1). The mean time between diagnosis and toceranib start was 11 days (range 2–19 days). Seven dogs received toceranib as a single agent. One received concurrent steroids.

Four dogs treated in the gross disease setting were evaluated for clinical benefit via imaging (Table 1). One achieved a partial response, 2 stable disease, and 1 had progressive disease, indicating a clinical benefit rate of 75 % (Table 1). An additional dog had clinically stable disease that was not confirmed via imaging. At the time of data collection, 1 dog (Case #2) with image-confirmed stable disease had developed disease progression with an overall toceranib-specific survival of 302 days. The other dog (Case #6) continued to have image-confirmed stable disease for 506 days since starting toceranib. An additional dog (Case #8) was assumed to have stable disease as no clinical signs attributable to pancreatic carcinoma developed while on toceranib; however, when the toceranib was discontinued, the patient clinically declined developing weight loss, weakness, and polyuria/polydipsia. The second dog clinically evaluated for response (Case #1) developed hyporexia, lethargy, and pain attributable to progressive disease before reaching the stable disease criteria of 10 weeks, and thus was classified as having progressive disease.

The patient with a partial remission (Case #5) experienced clinical benefit at least 41 days after starting toceranib. At that time, based on ultrasound evaluation, the pancreatic carcinoma (located in the right lobe of the pancreas) had decreased in size from 2.6 to 1.6 cm in longest diameter. In addition, previously noted lymphadenopathy had resolved, and multiple liver nodules suspected to be metastatic disease were stable. Unfortunately, 56 days after documentation of the partial response, progressive disease developed characterized by an increase in size of the pancreatic mass with invasion of the duodenum, appearance of an adjacent venous thrombus, pancreatic lymphadenopathy, progressive and more numerous liver nodules, and a small amount of peritoneal effusion for a toceranib-specific survival time of 97 days.

Of the two patients treated in the microscopic disease setting following surgery, one had progressive disease 14 days after starting toceranib; the second had no evidence of tumour regrowth when it was lost to follow-up at 82 days after starting toceranib.

Toceranib was generally well-tolerated. Adverse events were noted in 37 % of dogs and included grade I nausea (*n* = 1), grade 1 diarrhoea (*n* = 1), grade 2 diarrhoea (*n* = 1), grade 1 anorexia (*n* = 1), and grade II neutropenia (*n* = 1). Two patients required a toceranib holiday due to chronic diarrhoea and anorexia (*n* = 1) or recurrent grade II neutropenia (*n* = 1). A dose reduction alleviated further adverse events. During the study time frame, palladia was discontinued in 7/8 dogs (88 %). In four dogs, this was due to the development of progressive disease. In two dogs, the reasons were not specifically stated, but were not due to drug intolerance or owner’s wishes. In one dog, toceranib was discontinued due to owner wishes.

At study conclusion, 1 patient was alive, 5 had died/were euthanized, and 2 were lost-to-follow-up. Although no necropsies were completed, death in 4/5 dogs was attributed to the pancreatic carcinoma, while cause of death was unknown in the remaining patient (Table 1). The toceranib-specific median overall survival time for all patients was 89.5 days (range: 14–506 days).

## Discussion

Canine pancreatic carcinoma is a rare, aggressive cancer that is highly metastatic at presentation and resistant to chemotherapy. Beyond surgical removal, effective treatment regimens for dogs have remained elusive [3]. In cats and humans, gemcitabine chemotherapy appears to have some efficacy [14], as do TKIs [8, 15, 16].

In humans, upregulation of VEGFR is associated with a more severe clinical course of disease and an increased metastatic potential. Blockade of this receptor with sunitinib results in clinical response and benefit [8, 12]. Unfortunately, the VEGFR status in canine pancreatic carcinoma has not been investigated. However, other neuroendocrine tumours including thyroid carcinoma [17], apocrine gland anal sac adenocarcinoma [18], and pheochromocytoma [19] have been shown to express or overexpress VEGFR. Clinical benefit to toceranib, which blocks VEGFR, has also been implied for these cancers [20, 21]. This suggests that perhaps canine pancreatic tumours would have similar expression of these tyrosine kinase receptors, possibly explaining the biologic response to toceranib. Evaluation of the expression of VEGFR in a larger population of patients will be required in future studies to strengthen and confirm this argument.

Feline pancreatic carcinomas, which are biologically similar to both human and canine pancreatic tumours [14], also appear to have a biologic response to toceranib [15, 16]. Similar to dogs, VEGFR expression has not been evaluated in feline pancreatic carcinoma. However, increased expression of VEGFR has been shown in feline mammary carcinoma [22], along with preliminary evidence of biologic response to toceranib [23], strengthening the argument for toceranib use in both feline and canine pancreatic carcinoma.

In humans, exocrine pancreatic carcinoma is typically ductal in origin, while pancreatic acinar carcinoma is rare [24]. *KRAS* is a gene that regulates cellular signalling cascades associated with cellular growth, proliferation, and survival. Mutations in *KRAS* are common in human ductal carcinoma [25] and confer failure of sunitinib treatment in transgenic mouse models and possibly explain decreased response in some people to sunitinib [26]. These mutations are rarely reported in human acinar cell carcinoma and have recently been shown not to be present in canine acinar cell pancreatic carcinoma [27]. This suggests that inherent resistance to toceranib through *KRAS* mediated pathways should not impact response to toceranib in dogs with the more common acinar pancreatic carcinoma.

In this population of patients, toceranib was generally well tolerated. Adverse events were those commonly associated with toceranib and were not severe including low-grade anorexia, nausea, diarrhoea, and neutropenia [28–30]. No dogs experienced severe adverse events to warrant discontinuation of toceranib, although 2/8 (25 %) did require a drug holiday and dose reduction. This is similar to the percentage of patients requiring a drug holiday and/or dose reduction in a previous study evaluating the use of toceranib in various solid tumours [30]. In humans with pancreatic carcinoma, treatment with chemotherapy often improves quality of life even if a survival advantage is not attained or is insignificant [31]. As toceranib was well-tolerated in this canine population and conferred a clinical benefit rate of 75 %, treatment with toceranib could be considered regardless of documented response if improvement in quality of life is achieved. Additional, prospective studies will be required to adequately assess the impact of toceranib on both the clinical response and quality of life of canine patients with pancreatic carcinoma.

Due to the rarity of canine pancreatic carcinoma, patient numbers in this study are small, despite soliciting case information from all boarded oncologists and internists who receive the electronic ACVIM listserv. This small number limits statistical analysis, and interpretation and extrapolation of survival times reported here. Additional limitations are those inherent to a retrospective study including lack of standard evaluations and imaging modalities during toceranib treatment, clinically-based evaluation of response to treatment, confirmation of suspect metastatic disease, lack of histopathologic determination of acinar vs. ductal carcinoma, and lack of evaluation for VEGFR expression. It is also difficult to define the use of toceranib following cytoreductive surgery, as there were only 2 patients in this group with microscopic disease only.

## Conclusions

The findings of this small, retrospective analysis suggest that toceranib may have biologic activity for canine pancreatic carcinoma. Toceranib was well-tolerated and provided a clinical benefit rate of 75 % with a toceranib-specific median overall survival time of 89.5 days, certainly better than the previously published 8 days [3]. These results support the use and further investigation of toceranib for dogs with pancreatic carcinoma.

## Methods

Cases of canine pancreatic carcinoma treated with toceranib were retrospectively solicited via the ACVIM Internal Medicine and Oncology listservs using an electronic survey (REDCap, Vanderbilt University, Nashville, TN, USA). Dogs with a cytologic or histologic diagnosis of pancreatic carcinoma, treated with toceranib, were eligible for inclusion. Data collected included signalment (age, sex, breed, weight), presenting clinical signs, method of diagnosis, presence of metastatic disease, location and size of tumour in the longest diameter, toceranib dose (mg/kg), schedule and duration of treatment, best response, adverse events, concurrent chemotherapy and supportive medications, concurrent diseases, and cause of death, if known or applicable. Staging and follow-up intervals were at the discretion of the attending clinician and were variable. Best response to treatment was categorized as complete response, partial response, stable disease, or progressive disease based on the Response Evaluation Criteria for Solid Tumours v1.0 [32]. Adverse events were graded using the Veterinary Cooperative Oncology Group Common Terminology Criteria for Adverse Events v1.1 [33].

For dogs with measurable disease, clinical benefit was determined by best response to therapy and was defined as complete response, partial response, or stable disease > 10 weeks in duration, as has been previously described [20]. Toceranib-specific median overall survival time was calculated for all dogs from the first toceranib dose to progressive disease (defined as local regrowth, local progression, and/or evidence of new or progressive metastatic disease), discontinuation of toceranib, death while on toceranib, or last date of contact while on toceranib. Due to the sample size, descriptive statistical analysis was completed.

## Data Availability

The datasets used and/or analyzed during the current study are available from the corresponding author on reasonable request.

## Abbreviations

ACVIM: American College of Veterinary Internal Medicine
EGFR: Epithelial growth factor receptor
PDGF/PDGFR: Platelet-derived growth factor/receptor
TKI: Tyrosine kinase inhibitor
VEGF/VEGFR: Vascular endothelial growth factor/receptor
